# ΔNp63α and microRNAs: leveraging the epithelial-mesenchymal transition

**DOI:** 10.18632/oncotarget.13797

**Published:** 2016-12-04

**Authors:** Andrew J. Stacy, Michael P. Craig, Suraj Sakaram, Madhavi Kadakia

**Affiliations:** ^1^ Department of Biochemistry and Molecular Biology, Wright State University, Dayton, OH, USA

**Keywords:** EMT, p63, miRNA, signaling, biomarker

## Abstract

The epithelial-mesenchymal transition (EMT) is a cellular reprogramming mechanism that is an underlying cause of cancer metastasis. Recent investigations have uncovered an intricate network of regulation involving the TGFβ Wnt, and Notch signaling pathways and small regulatory RNA species called microRNAs (miRNAs). The activity of a transcription factor vital to the maintenance of epithelial stemness, ?Np63a, has been shown to modulate the activity of these EMT pathways to either repress or promote EMT. Furthermore, ?Np63a is a known regulator of miRNA, including those directly involved in EMT. This review discusses the evidence of ?Np63a as a master regulator of EMT components and miRNA, highlighting the need for a deeper understanding of its role in EMT. This expanded knowledge may provide a basis for new developments in the diagnosis and treatment of metastatic cancer.

## INTRODUCTION

Cancer cell metastasis is the leading cause of death from cancer, however, the cellular mechanisms of metastasis remain incompletely characterized [1]. The process by which carcinoma cells can become migratory and invasive is proposed to occur via EMT, a cellular reprogramming mechanism by which epithelial cells acquire a motile mesenchymal phenotype, leading to the migration of cells to nonadjacent target sites [2, 3]. Once at these target sites these cells will regain an epithelial phenotype via a process called the Mesenchymal-Epithelial transition (MET) [4]. Epithelial cells are characterized by cell-cell adhesion, non-motility as a result of extracellular matrix anchorage, and an apical-basal polarity [2]. Mesenchymal cells typically lack cell-cell adhesion, degrade the extracellular matrix to become motile and invasive, display apoptotic resistance, and exhibit a lack of polarity [5]. EMT is essential for developmental processes such as gastrulation, neural crest formation, and wound healing; however, EMT can also lead to pathological states, such as organ fibrosis and tumor cell metastasis [6].

Many epithelial markers, including tight junction proteins such as zona occludens-1 (ZO-1), occludins, and the claudins, as well as the adherens junction protein epithelial cadherin (E-cadherin), are repressed during EMT [7]. The most frequently observed predictive harbinger of EMT progression is the downregulation of E-cadherin [8]. Mesenchymal cells instead express neural cadherin (N-cadherin), vimentin, an intermediate filament protein that is vital to mesenchymal organelle cytoskeletal arrangement, and fibronectin, a glycoprotein that functions in migration [9]. Promotion of EMT is accomplished primarily by the transcription factors TWIST, SNAIL, SLUG, and ZEB [8]. SNAIL1 and SLUG (also known as SNAIL2) bind to and repress the E-cadherin promoter CDH1 and trigger the switch to N-cadherin [10, 11]. TWIST is known to result in downregulation of E-cadherin and promote EMT through upregulation of SLUG [12]. ZEB1 and ZEB2 (ZEB1/2) also repress E-cadherin and upregulate matrix metalloproteinases (MMPs) to degrade the extracellular matrix, which allows for increased cellular mobility [13, 14].

An additional transcription factor recently shown to inhibit EMT is p63, a member of the p53 family. The p63 gene encodes six primary isoforms through differential promoter usage and C-terminal splicing [15]. The alternative promoters result in two classes of p63 constituting either a full-length transactivation domain at the N-terminus, designated TAp63, or a truncated N-terminus lacking the transactivation domain, designated ΔNp63 [16]. Alternative splicing of TAp63 and ΔNp63 create distinct C-termini, designated α, β, and γ. All of these isoforms contain a DNA binding domain and an oligomerization domain; however, the α isoforms also contain a sterile alpha motif (SAM) protein-protein binding domain and a trans-inhibitory domain [17]. This review primarily focuses on the most physiologically relevant isoform, ΔNp63α, unless otherwise noted. ΔNp63α is highly expressed in the basal layer of epithelia where it plays a role in cellular proliferation and is downregulated in suprabasal keratinocytes [16]. p63 is vital to epithelial morphogenesis, as p63 null mice are born lacking limbs or stratified epithelium resulting in their death shortly following birth due to dehydration [18]. Additionally, p63 knockout mice have no hair or teeth, and exhibit defects in mammary gland development [19].

ΔNp63α has a vital role in the inhibition of EMT and promotion of the epithelial phenotype. ΔNp63α downregulates mesenchymal genes and simultaneously upregulates epithelial genes, particularly those involved in cell adhesion, such as Claudin1, and integrins involved with cellular adhesion to the extracellular matrix [20–22]. ΔNp63α also induces the expression of the transcription factor inhibitor of differentiation-3 (ID3), which inhibits the expression of both an E-cadherin transcriptional repressor, E2A, and an extracellular matrix degradation enzyme, MMP2 [23]. Inhibiting ΔNp63α upregulates genes that promote mesenchymal morphology and motility, such as N-cadherin, leading to increased cell invasion and metastasic potential [24]. ΔNp63α is transcriptionally repressed by Snail1, thus suggesting that p63 is also downstream of known EMT-related transcription factors [25]. Loss of ΔNp63α can also lead to a reduction in MET [26].

## ΔNP63α INVOLVEMENT WITH EMT SIGNALING PATHWAYS

**Figure 1:**
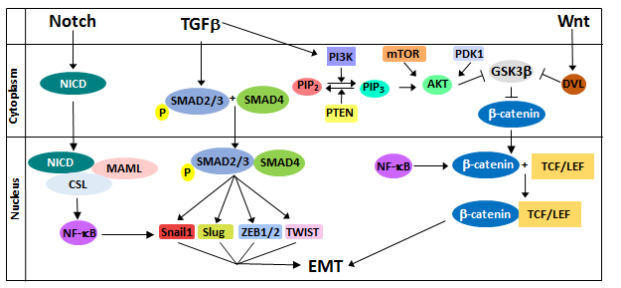
Regulation of EMT by the Notch, TGFβ, and Wnt signaling pathways Notch signaling results in the transcriptional upregulation of NF-κB, which induces EMT by upregulating SNAIL1 and by inducing stabilization of β-catenin. TGFβ activates SMAD proteins to promote transcription of the EMT transcription factors SNAILl, SLUG, ZEB, and TWIST. Additionally, TGFβ crosstalk with the Wnt pathway occurs through the PI3K/AKT pathway. Wnt signaling stabilizes β-catenin expression, allowing it to form a transcriptional complex with TCF/LEF to upregulate EMT regulators such as Snail, Slug, and Vimentin.

**Figure 2:**
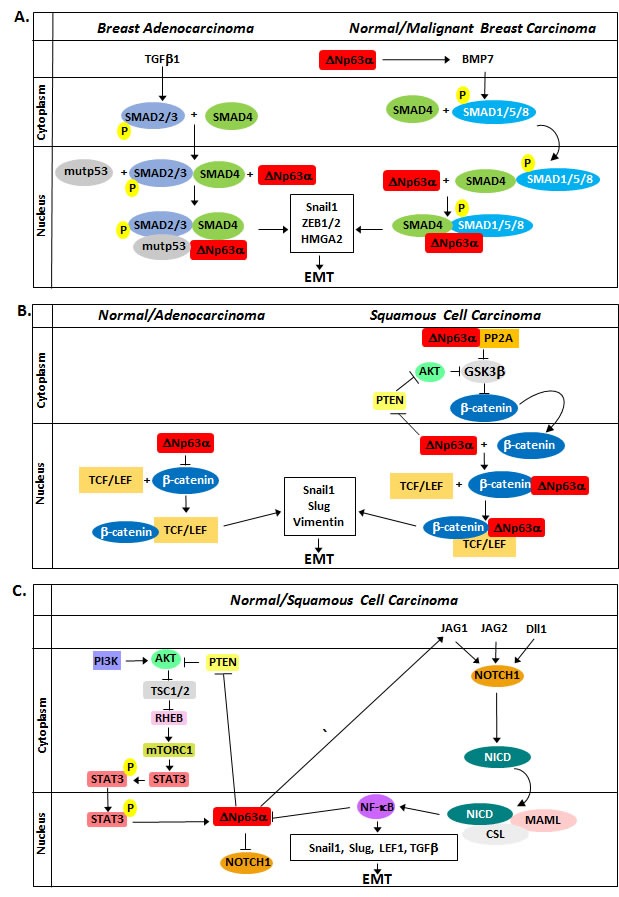
Role of ΔNp63α in the modulation in EMT with regard to the TGFβ, Wnt, and Notch pathways A. TGFβ1 and BMP7 signaling results in SMAD phosphorylation and formation of a transcriptional complex with SMAD4 and ΔNp63α to induce EMT. Additionally, ΔNp63α increases TGFβ signaling by upregulating BMP7 transcription. B. ΔNp63α elicits seemingly contradictory effects on Wnt signaling. In normal keratinocytes and adenocarcinoma cell lines, ΔNp63α represses Wnt signaling by competing with β-catenin for Wnt response elements. In invasive carcinoma lines, ΔNp63α promotes β-catenin stability, potentially by inhibiting PTEN and promoting AKTkt phosphorylation and activation, and forms a transcription complex with β-catenin and TCF/LEF to promote EMT. C. PI3K/AKT signaling results in the activation of STAT3 and increased ΔNp63α expression. ΔNp63α then acts through Jagged1 to induce Notch signaling in adjacent cells, leading to increased NF-κB expression and inhibition of ΔNp63α expression to promote EMT.

Multiple signaling pathways, including Transforming Growth Factor β (TGFβ), Wnt, and Notch, are involved in the cellular regulation of TWIST, SNAIL, SLUG, and ZEB. The representative signaling cascades are shown in Figure 1 [8]. Inhibition of these pathways, their components, or their target genes can repress EMT [27–30]. The interplay between these pathways and ΔNp63α is dynamic. The evidence presented here indicates that several pathways modulate ΔNp63α expression and activity to induce rather than inhibit EMT. The involvement of ΔNp63α in these pathways is summarized in Figure 2.

## TRANSFORMING GROWTH FACTOR β SIGNALING

The TGFβ pathway regulates multiple cellular processes, such as proliferation, differentiation, cytoskeletal rearrangements, metastasis, and apoptosis [31]. Signaling is activated by the TGFβ ligand cytokine superfamily (TGFβ1/2/3) and Bone Morphogenic Protein 2 (BMP2)-BMP7, with TGFβ1 being the principal driver of EMT [31]. Binding of a TGFβ ligand to its receptor induces SMAD2/3 phosphorylation, resulting in a complex with SMAD4 and translocation to the nucleus to target genes for transcription [32]. SMAD signaling leads to the upregulation of SNAIL1, ZEB1/2, and the non-histone chromatin binding protein high mobility group A2 (HMGA2) [33]. HMGA2 forms a complex with SMAD3-SMAD4 to enhance SNAIL1, SLUG, and TWIST transcription to promote EMT [34].

SMAD proteins alone bind DNA weakly and, as a functional necessity, associate with other transcriptional factors to increase their binding affinity, including ΔNp63α (Figure 2A). Signaling induced by TGFβ1 drives formation of a complex consisting of ΔNp63α, mutant p53, and SMAD2, which results in the repression of ΔNp63α transcriptional activity while promoting SMAD2 activity, resulting in increased invasiveness and metastasis [35]. ΔNp63α also transcriptionally induces BMP7 in breast tumors [36]. SMADs1/5/8, activated by BMP7, have been shown to interact with ΔNp63α to regulate BMP transcriptional targets, including SNAIL [37]. Other reports further support ΔNp63α as a SMAD co-factor, because ΔNp63α in the presence of TGFβ signaling silences E-cadherin while increasing fibronectin expression [38]. It can therefore be concluded that SMAD proteins interact with and alter the activity of ΔNp63α, resulting in promotion rather than inhibition of EMT. Supporting this conclusion is the observation that re-expressing ΔNp63α in mesenchyme-like cells does not completely restore the epithelial phenotype [21]. This evidence suggests that the SMAD-ΔNp63α complex competes with free ΔNp63α for access to response elements and explains why reintroduction of ΔNp63α in the presence of TGFβ signaling does not fully induce an epithelial phenotype. This raises the hypothesis that EMT signaling pathways can modulate ΔNp63α activity to promote EMT, as ΔNp63α has also been observed to promote EMT in mammary and other tissues [39, 40].

## WNT SIGNALING

Extracellular matrix-associated Wnt ligands bind to Frizzled and low-density lipoprotein receptor-related protein (LRP) receptors to initiate Wnt signaling. In the absence of Wnt ligand activation, β-catenin is phosphorylated by glycogen synthase kinase-3β (GSK3β), which is in complex with AXIN and adenomatosis polyposis coli (APC). This phosphorylation ultimately targets β-catenin for proteasomal degradation. Induction of Wnt signaling results in activation of Dishevelled (DVL), which recruits GSK3β and Axin to the plasma membrane and preventing GSK3β from phosphorylating β-catenin [41]. This leads to the accumulation of β-catenin and its translocation to the nucleus, where it complexes with the T cell factor (TCF)/Lymphocyte enhancer factor (LEF) transcription factors to induce the transcription of EMT components such as vimentin, SNAIL1, and SLUG [41]. Wnt signaling can participate in crosstalk with TGFβ signaling since β-catenin, as well as SMAD2/3 in complex with SMAD4, can activate TCF/LEF [9]. Intriguingly, most colorectal tumors exhibit an increase in intracellular β-catenin yet do not display mesenchymal features [5]. This indicates that β-catenin acts as a co-activator for the expression of EMT transcription factors but cannot induce them alone. Thus, β-catenin is a molecular node providing crosstalk between Wnt and other EMT signaling pathways, including the TGFβ and Notch pathways.

There are contradictory observations regarding the role of ΔNp63α within the Wnt pathway (Figure 2B). It has been observed that ΔNp63α decreases β-catenin phosphorylation to allow for β-catenin nuclear accumulation in Saos-2 osteosarcoma cells, where ΔNp63α/β-catenin can then associate to promote TCF/LEF to induce upregulation of mesenchymal biomarkers vimentin and Snail [42]. A second study using non-small cell lung carcinoma cells (H1299) and human embryonic kidney cells (HeK293) cells found that ΔNp63α opposes Wnt signaling and inhibits Wnt downstream targets, thus suggesting the observed contradictions of ΔNp63α-modulation of Wnt signaling may be due to differing experimental conditions [43]. Endogenous ΔNp63α was found to induce vimentin, SNAIL, and TWIST in esophageal squamous carcinoma cell lines to promote migration/invasion in a β-catenin-dependent manner, while showing little to no effect on EMT biomarkers in esophageal adenocarcinoma cell lines [44]. Supporting this observation, ΔNp63α was found to activate Wnt target genes in a squamous cell carcinoma cell line (FaDu), while repressing Wnt response elements in HEK293 cells [45]. A potential explanation for the positive regulation of β-catenin by ΔNp63α could depend on the status of AKT activation. ΔNp63α is a negative transcriptional regulator of PTEN, promoting AKT phosphorylation and activity [46]. AKT subsequently phosphorylates GSK3β, leading to its inactivation and allowing for β-catenin nuclear accumulation [47]. However, it appears that ΔNp63α can also compete with β-catenin for binding to Wnt response elements to inhibit Wnt signaling [45]. This contradiction highlights the need for further investigation. Together these results suggest that invasive carcinomas can influence the activity of ΔNp63α to promote a mesenchymal phenotype, either by Akt signaling or potentially even through a tertiary co-factor such as a SMAD or mutant p53, which could associate with ΔNp63α/β-Catenin.

## NOTCH SIGNALING

The Notch pathway is involved in cellular proliferation, differentiation, apoptosis, and survival [48]. Notch signaling is initiated when a cell expressing a Notch transmembrane receptor (NOTCH1-4), consisting of an extracellular and intracellular (NICD) domain, comes into contact with another cell expressing one of the transmembrane Notch ligands, including Delta-like ligand 1/3/4 (DLL1/3/4) and Jagged1/2 [49]. This interaction induces the proteolytic cleavage of NOTCH to form the NICD. Once cleaved, the NICD translocates to the nucleus where it interacts with the DNA-bound transcriptional repressor CBF-1-Suppressor of Hairless/Lag1 (CSL) [49]. This complex displaces corepressors and recruits the co-activating Mastermind-like protein (MAML) to form the Notch-CSL-MAML complex [50]. The NOTCH-CSL-MAML complex subsequently recruits members of the Notch transcriptional complex to activate gene expression [51]. For example, Notch signaling upregulates Nuclear Factor-κB (NF-κB) [5, 8]. NF-κB can induce EMT by upregulating Snail1, Slug, and Lef-1, as well as by stabilizing β-catenin and modulating TGFβ signaling activity [5, 8].

Cells expressing ΔNp63α can induce Notch signaling on adjacent cells while simultaneously repressing this pathway within themselves. This is due in part to crosstalk between NOTCH and the PI3K-AKT pathway (Figure 2C). As mentioned in the previous section, ΔNp63α can promote AKT phosphorylation by inhibiting PTEN expression [46]. AKT leads to the activation of mTORC1, which phosphorylates STAT3 to induce its translocation to the nucleus. STAT3 then upregulates ΔNp63α, which influences Notch signaling by inducing the expression of the Notch ligand Jagged1. Jagged1 then induces Notch signaling in adjacent cells. Additionally, there is negative feedback between ΔNp63α and Notch pathway components. DLL1 and Jagged1 ligands signal through the NOTCH1 receptor, inducing NF-κB, which then inhibits the activity and promotes the proteasomal degradation of ΔNp63α by a poorly understood mechanism in squamous cell carcinoma and non-small cell lung cancer [52]. Conversely, ΔNp63α inhibits Notch-dependent transcription and represses NOTCH1 receptor expression, indirectly inhibiting the activity of NF-κB [53]. The relationship between Notch signaling and ΔNp63α is important to the establishment of the ectoderm, as ΔNp63α expression is lost through the suprabasal layers with increased Notch activity [54]. However, the Notch-ΔNp63α relationship has potential implications in a tumor microenvironment with heterogeneous ΔNp63α expression. Cells overexpressing ΔNp63α can express Jagged1 and stimulate NF-κB through NOTCH1 in adjacent cells expressing little to no ΔNp63α to potentially promote EMT [55]. This could also help explain contradictory reports with regard to EMT correlated with ΔNp63α.

In conclusion, ΔNp63α is involved with components of signaling pathways found to induce EMT. Therefore, there is a clear connection established between ΔNp63α and the cellular regulation of EMT.

## MICRORNAS THAT INHIBIT EMT

miRNAs are small non-coding RNAs shown to target EMT transcription factors, as shown in Table 1 [56]. miRNA are approximately 17-23 nucleotides in length and inhibit gene expression by preventing translation of target mRNA. miRNA are transcribed from the genome and processed by the RNase III endonuclease DROSHA [57]. Alternatively, miRNA known as Mirtrons are transcribed from the introns of genes and do not require processing by DROSHA [58]. Both the intrinsic canonical and intronic pre-miRNA are then transported to the cytoplasm to be processed by the RNase III enzyme DICER [57]. The miRNA are then loaded into a multi-protein complex, the RNA Induced Silencing Complex (RISC). miRNAs contain a 2-8 nucleotide seed sequence, called the guide strand, which recognizes a complementary sequence in the 3’-UTR region of target mRNA, resulting in the degradation or translational repression of the target transcript [57]. Multiple mRNAs may be targeted by a single miRNA. This includes EMT transcription factors, invasion and migratory proteins, regulators of miRNA biosynthesis such as DROSHA and DICER, and the primary focus of this review, ΔNp63α.

**Table 1:** miRNAs shown to inhibit or promote EMT

miRNAs	Target(s)	Reference
Let-7	HMGA2	[65]
1	SLUG	[68]
9	NF-κB, E-cadherin	[90, 91]
10b	HOXD10	[92, 93]
29b	SNAIL1	[79]
30	SNAIL1	[80]
34a, 34b, 34c	ZEB1, SNAIL1, SLUG	[78]
130b	ZEB1	[74]
138	ZEB2	[76]
192	ZEB2	[125]
141, 200a, 200b, 200c, 429	ZEB1/2, β-catenin	[71, 72]
203	SNAIL1, SLUG	[69, 81]
204	TGFBRII	[67]
205	ZEB1/2	[61]
221, 222	TRPS1	[89]
365	HMGA2	[64]
455-3p	RUNX2	[87]

ΔNp63α has been implicated in the direct transcriptional control of miRNA responsible for regulating many cellular processes, including EMT (Tables 2 and 3) [59]. For example, ΔNp63α promotes the expression of miR-205 by binding directly to its promoter and recruiting RNA polymerase II [60]. This identifies ΔNp63α as a negative regulator of EMT, as miR-205 in turn targets ZEB1/2 [60, 61]. Loss of ΔNp63α and miR-205 have also been correlated to poor clinical outcomes in patients [59]. In addition to EMT transcription factors, miRNAs regulated by ΔNp63α target portions of EMT signaling pathways. The TGFβ pathway can be targeted by multiple miRNA regulated by ΔNp63α, including miR-155, which targets SMAD2 to attenuate TGFβ signaling [62, 63]. ΔNp63α has also been observed to regulate other miRNA that potentially promote EMT, as discussed in the ‘MicroRNAs that promote EMT’ section.

**Table 2:** miRNAs upregulated by p63

miRNA (miR-)	Target Gene(s)	Function	Cell/Tissue Type	Detection method	Reference
17, 106a	MAPK1 (Erk2),p21,RB and MAPK9 (JNK2)	Regulation of keratinocyte differentiation	HaCaT	1,2	[86]
18a	HIF-1α	Regulation of keratinocyte differentiation	HaCaT	1,2	[86, 126]
20b	MAPK1,p21 and MAPK9 (JNK2)	Regulation of keratinocyte differentiation	HaCaT	1,2	[86]
30a	NFATc3, LOX	Regulation of keratinocyte differentiation	HaCaT	1,2	[86, 127, 128]
92b-3p	HDAC9, KAT2B, ATOX1, CDKN1C	Epigenetic Regulation, Cell Metabolism, Cell Cycle Arrest, Apoptosis	SCC-11	3	[59]
143	MAPK1 (Erk2)	Regulation of keratinocyte differentiation	HaCaT	1,2	[86]
155	HIF-1α, FADD, CASP3, SMAD2	Cell Migration, Tumor Growth	MCF10a, A431	2	[63]
185-5p	ATF6, DNMT1, SREBF2, SREBF, FADS1, HMGCR, CASP2, CASP14, PARP11	Epigenetic Regulation, Cell Metabolism, Cell Cycle Arrest, Apoptosis	SCC-11	3	[87]
194-3p	GRABARAPL1	Autophagy	SCC-11	3	[87]
194-5p	KAT6B, SIRT1, ATM, CASP7	Epigenetic Regulation, Cell Cycle Arrest, Apoptosis	SCC-11	3	[88]
205	ZEB1 and ZEB2	EMT regulation	UC3, UC6	2,4	[60]
297	DNMT3A, SIRT3, SKP2, ATM, ATP7A, ATG5	Epigenetic Regulation, Cell Metabolism, Autophagy	SCC-11	3	[88]
382-3p	NFYB, ETNK, CDK1	Epigenetic Regulation, Cell Metabolism, Cell Cycle Arrest, Apoptosis	SCC-11	3	[88]
455-3p	MAPK8 (JNK1)	Regulation of keratinocyte differentiation	HaCaT	1,2	[86]
485-5p	KDM4C, ETNK, H6PD, PARP8, DFFA	Epigenetic Regulation, Cell Metabolism, Cell Cycle Arrest, Apoptosis	SCC-11	3	[59]
610	ATF5	Epigenetic Regulation	SCC-11	3	[85]
630	EZH2, KAT3B, ZBTB2, UVRAG, ATG2B, ATG4C, ATG12	Epigenetic Regulation Autophagy	SCC-11	3	[87]
637	ATF3	Epigenetic Regulation	SCC-11	3	[85]
760	BMF	Cell Cycle Arrest, Apoptosis	SCC-11	3	[85]
885-3p	CARM1, AKT1, CASP3, ULK2, ATG16	Epigenetic Regulation, Cell Metabolism, Cell Cycle Arrest, Apoptosis, Autophagy	SCC-11	3	[26]
920	KAT6B, NFYB	Epigenetic Regulation	SCC-11	3	[85]

Detection method: 1: miRNA-microarray, 2: qPCR, 3: miRNA-Chip microarray, 4: ChIP

**Table 3:** miRNAs downregulated by p63

miRNA (miR-)	Target Gene(s)	Function	Cell/Tissue type	Detection Method	References
7a-5p	CASP3, XIAP	Cell Cycle Arrest, Apoptosis	SCC-11, cervical cancer	3	[85]
18a-5p	CPS1, CPS2 (CAD), CASP7	Cell Metabolism, Cell Cycle Arrest, Apoptosis	SCC-11	3	[85]
22-3p	KDM3A, KAT6B, SIRT1, MECP2, ATG2B	Epigenetic Regulation, Autophagy	SCC-11	3	[88]
25-3p	HDAC9, CDK1C	Epigenetic Regulation, Cell Cycle Arrest, Apoptosis	SCC-11	3	[88]
27a-3p	HDAC9, KDM3A, p53	Epigenetic Regulation	SCC-11	3	[59]
29c-3p	DNMT3B, KDM2A, HDAC4, SIRT1, CPS1, AKT2, BMF, CDK2	Epigenetic Regulation, Cell Metabolism, Cell Cycle Arrest, Apoptosis	SCC-11	3	[88]
34c-3p	BMI1,EED, DNMT1, BMF, ATG4C, DRAM1	Epigenetic Regulation, Cell Cycle Arrest, Apoptosis, Autophagy	SCC-11	3	[88]
98-5p	CASP3, ATG10	Cell Cycle Arrest, Apoptosis, Autophagy	SCC-11	3	[88]
101a-3p	EZH2, DNMT3A, COX2, AKT1, ATG4D, RAB5A	Epigenetic Regulation, Cell Metabolism, Autophagy	SCC-11	3	[88]
130b	ΔNp63α	Senescence	HEKn	1,2	[94]
138, 181a/b	SIRT-1	Senescence	HEKn	1,2	[94]
148a-3p	DNMT1, DNMT3B	Epigenetic Regulation	SCC-11, breast and gastric cancers	3	[88]
155-5p	SP3, KDM2A, KDM5B, APAF1, GABARAPL1	Epigenetic Regulation, Cell Cycle Arrest, Apoptosis, Autophagy	SCC-11	3	[88]
181a-5p	HDAC4, SIRT1, KAT2B, ATM, ATG, p63	Epigenetic Regulation, Autophagy	SCC-11	3	[87]
183-5p	RNF5, KDM3A, KDM5B, ATG12	Epigenetic Regulation, Autophagy	SCC-11	3	[85]
193a-5p, 602, 765	p73	Proliferation, apoptosis	JHU-029 SCC; p63lox mice	1,2,3	[129]
203a	NFYA, CITED2, KAT6B, ATM, ATP7B, CPS1, FADS1, ATG2B, GABARAPL1, p63	Epigenetic Regulation, Cell Metabolism, Autophagy	SCC-11	3	[88]
206	CITED, KAT6A	Epigenetic Regulation	SCC-11	3	[88]
339-3p	DNMT3B, GABARAPL1	Epigenetic Regulation, Autophagy	SCC-11	3	[88]
362-3p	SIN3A, E2F1	Epigenetic Regulation	SCC-11	3	[85]
374a-5p	SP1, NFYB, CRTC2, KAT2B, ATM, ATG4A, ATG4A, ATG5, UVRAG, p63	Epigenetic Regulation, Autophagy	SCC-11	3	[46]
429	CITED2, E2F3, NFYA, CASP2, CDKN2B, CDK2, BCL2	Epigenetic Regulation, Cell Cycle Arrest, Apoptosis	SCC-11	3	[88]
485-3p	MAPILC3B	Autophagy	SCC-11	3	[85]
519a-3p	KDM2A, KDM5B, BHLHE31, ATM, CASP2, CDKN2B, ATG10, ATG16L1, UVRAG, p63	Epigenetic Regulation, Cell Cycle Arrest, Apoptosis, Autophagy	SCC-11	3	[46]
527	TGFβRII, SMAD4	Wound Healing, Migration	JHU-029, MNNG/HOS	1, 2	[95]
665	TGFβRII, SMAD4	Wound Healing, Migration	JHU-029, MNNG/HOS	1, 2	[95]

Detection method: 1: miRNA-microarray, 2: qPCR, 3: miRNA-Chip microarray

TGFβ signaling is targeted by additional miRNAs, such as the Let-7 miRNA family in human pancreatic cells, and by miR-365 in lung adenocarcinoma [64, 65]. These miRNAs additionally target HMGA2, indirectly reducing Snail1 expression [66]. A TGFβ receptor, TGFβRII, is inhibited by miR-204. miR-204, miR-1, and miR-203 can directly target SLUG, inhibiting the pathway [67]. Interestingly, SLUG also inhibits the expression of miR-1 and miR-203, in a positive feedback manner [68, 69]. This directly demonstrates a regulatory network between EMT signaling pathways and miRNA.

Supporting a role for miRNA in epithelial integrity, expression levels of E-cadherin are positively correlated with those of miR-205 and the miR-200 miRNA family, which also target ZEB1/2 [70]. The miR-200 family (henceforth referred to as miR-200) consists of five members, including miR-200a, miR-200b, miR-429, miR-200c and miR-141 [71]. Accompanying the suppression of ZEB 1/2, miR-200 can also target β-catenin to interrupt EMT signaling [72]. In turn, ZEB1/2 can additionally inhibit transcription of miR-200 family miRNAs, thus there is reciprocal inhibition between ZEB 1/2 and miR-200 [73]. Other miRNAs target individual ZEB isoforms, with ZEB2 targeted by miR-138 and miR-192 [74–76], while miR-130b and the miR-34 family, consisting of miR-34a, miR34b, and miR-34c, downregulate ZEB1 expression [74, 77].

The miR-34 family (henceforth referred to as miR-34) also represses EMT through inhibition of SNAIL1 and SLUG expression [78]. Furthermore, while miR-34 can repress SNAIL1, SNAIL1 directly blocks miR-34 transcription, again displaying the reciprocal inhibition that characterizes EMT regulatory pathways [78]. In addition to miR-34, multiple miRNA negatively regulate SNAIL1, such as miR-29b, miR-30, and miR-203, with miR-203 shown to participate in a reciprocal inhibitory loop with Snail1 in breast cancer [79–81]. Interestingly miR-203 also targets ΔNp63α in differentiated keratinocytes demonstrating a potential cell type-specific response [82], with this relationship explored further in the regulation of ΔNp63α by miRNAs section.

Expression of several other miRNAs have also been found to correlate with ΔNp63α expression, and in silico analysis of these miRNAs identified p63 response elements in their promoters. These miRNAs include Let-7, miR-23, miR-29, miR-134, miR-145, miR-192, and miR-215 [83]. In addition to directly targeting miRNA for transcriptional regulation, the p63 gene encodes a Mirtron, miR-944. This miRNA targets transcripts involved in cellular proliferation, migration, and invasion [84]. Moreover, ΔNp63α has also been demonstrated both to upregulate miR-455-3p in an immortalized human keratinocyte cell line and to downregulate it in squamous cell carcinoma, demonstrating potential tissue-specific control of miRNA expression [85, 86]. miR-455-3p targets the RUNX2 transcript to inhibit EMT-promoting components, potentially indicating that ΔNp63α modulation by EMT signaling pathways may also affect miRNA expression profiles [87, 88].

## MICRORNAS THAT PROMOTE EMT

Certain miRNAs target transcripts of ZEB repressors, stabilizing ZEB expression. For example, miR-221 and miR-222 target the ZEB repressor TRPS1, resulting in E-cadherin downregulation to promote EMT [89]. Additional miRNAs, such as miR-9, can directly target and regulate E-cadherin expression. miR-9 is upregulated by the oncogenes MYC and MYCN, which are often overexpressed in metastatic breast cancer [90]. In a demonstration of tissue-specific activity to induce EMT, miR-9 has also been found to indirectly increase the expression of E-cadherin by targeting NF-κB in melanoma cells, inhibiting Notch signaling and thus acting as a tumor suppressor by indirectly suppressing the expression of SNAIL1 [91]. By contrast, miRNA-10b is known to promote tumor cell invasiveness and is induced by TWIST1. The metastatic action of miR-10b is a result of its inhibition of HOXD10 expression, a transcriptional inhibitor of proteins involved in cell migration and remodeling of the extracellular matrix such as the pro-metastatic protein Rho-Associated, Coiled-Coil Containing Protein Kinase (ROCK) and MMP14 [92, 93].

With miRNAs clearly involved in dual regulation of EMT, miRNAs regulated by ΔNp63α also share this dichotomy. Interestingly, ΔNp63α has been found to directly downregulate miRNA involved in the suppression of EMT, such as miR-130b and miR-138. [94]. The TGFβ pathway is promoted by ΔNp63α inhibition of miR-527 and miR-665, resulting in increased TGFβRII and SMAD4 expression [95].

As there are a large number of transcripts involved in EMT regulated by miRNA, regulation of microprocessors such as DICER and DROSHA is integral to the procession of EMT. DICER may be a transcriptional target of p63, as expression of DICER has been found to be influenced by the expression of TAp63. The promoters of several microprocessor components, DROSHA, DGCR8, DICER Dicer, and TARBP2, have also been found through computational analysis to have multiple p63 response element sites, with DGCR8 experimentally confirmed to be induced by ΔNp63α [96, 97].

## REGULATION OF ΔNP63α BY MICRORNAS

ΔNp63α itself is a target of multiple miRNAs. miR-203 targets the 3’-UTR of ΔNp63α to decrease cellular proliferation, resulting in repression of epithelial stemness [82]. The miR-203 promoter is hypermethylated in certain cancers and restoration of its expression has been demonstrated to downregulate p63, reduce migration and proliferation in vivo in a Notch dependent manner [98, 99]. miR-92a has similarly been shown to inhibit p63 [100]. Table 4 lists miRNAs validated to downregulate ΔNp63α.

**Table 4:** miRNAs that downregulate p63

miRNA (miR-)	Target Gene(s)	Function	Cell/Tissue type	Detection Method	Reference
130b	ΔNp63α	Senescence	HEKn	1, 2	[94]
20a-5p	p63	Glycogen synthesis	NCTC1469, Hep1-6	2	[130]
181a-5p	HDAC4, SIRT1, KAT2B, ATM, ATG5, p63	Epigenetic Regulation, Autophagy	SCC-11	3	[87]
196a2	TAp63	Proliferation	MCF-7, MDA-MB-231	1,2	[131]
203	ΔNp63	Epithelial Differentiation, Apoptosis	HEK 293E, NHEK, Primary Mouse Keratinocytes	2	[82]
203a	NFYA, CITED2, KAT6B, ATM, ATP7B, CPS1, FADS1, ATG2B, GABARAPL1, p63	Epigenetic Regulation, Cell Metabolism, Autophagy	SCC-11	3	[88]
223-5p	p63	Cell Migration, Invasion	SW962	2	[132]
92a	p63	Apoptosis, Proliferation	32D, HaCaT, HCT-116-Dicer-KO-2, HL-60	2	[133, 134]
301a	p63	EMT	PC3, LNCaP	2	[135]
374a-5p	SP1, NFYB, CRTC2, KAT2B, ATM, ATG4A, ATG5, UVRAG, p63	Epigenetic Regulation, Autophagy	SCC-11	3	[46]

Detection method: 1: miRNA-microarray, 2: qPCR, 3: miRNA-Chip microarray

Conversely, other miRNAs have either been demonstrated or have the potential to increase ΔNp63α expression. miR-145 is known to increase the expression of p63 through a mechanism yet to be identified [101]. miRNAs that target inhibitors of ΔNp63α, such as the E3 ubiquitin ligases that target ΔNp63α for proteasomal degradation, have the potential to upregulate ΔNp63α and thereby influence EMT. miR-106b targets ITCH, an E3 ubiquitin ligase that targets ΔNp63α [102, 103]. This is intriguing, as miR-106b has a role in the inhibition of EMT by also targeting TWIST1 [104]. F-box and WD repeat domain-containing 7 (FBW7), another E3 ligase that promotes ΔNp63α degradation, is targeted by miRNAs linked to EMT progression, miR-27a and miR-223 [105–109]. WW domain-containing E3 ubiquitin protein ligase 1 (WWP1), another inhibitor of ΔNp63α expression, is inhibited by miR-21 [110]. miR-21 expression has been correlated with increased p63 expression, and promotes EMT by increasing TGFβ signaling [110, 111]. This relationship is an additional link between miRNAs and EMT promotion through modulation of p63 and EMT signaling. Thus, there is evidence for the potential of miRNA expression patterns to predict the activity of ΔNp63α in the regulation of EMT. It is therefore of value to further investigate the co-regulation of ΔNp63α and miRNAs for the purpose of governing EMT.

## CLINICAL RELEVANCE

Due to the role of EMT in the development of key characteristics of metastatic cancer, including increased motility and resistance to apoptosis, there is therapeutic value in researching regulators of EMT. In cancer, there is often a decrease in expression levels of epithelial biomarkers which lead to increased tumor invasiveness and metastasis. For example, a model of pancreatic β-cell carcinogenesis reveals a link with the loss of E-cadherin and a switch from noninvasive adenoma to invasive carcinoma [112]. Additionally, there is an upregulation of Snail and vimentin found in aggressive breast cancers [113, 114]. Furthermore, p63 is often lost in invasive cancers associated with poor patient prognosis [115]. As evidenced by this review, there is a link between miRNAs and ΔNp63α co-regulation with the progression of EMT. Understanding the ΔNp63α-miRNA network would provide crucial information for diagnosis and treatment of EMT-governed tumor metastasis.

miRNAs are increasingly being investigated as biomarkers for premalignant lesions [116]. This is crucial within the context of this review, as patients with elevated expression of EMT biomarkers have a poor prognosis [117]. Along with an increase in metastasis as a result of the loss of E-cadherin observed in patients suffering from colon and prostate cancers, there is also increased expression of SLUG and TWIST1, and decreased expression of E-cadherin associated with increased breast cancer relapse [118–120]. Uncovering the regulation of miRNAs by ΔNp63α involved in EMT progression will therefore help predict tumor progression and patient outcome. For instance, high expression levels of miR-21 and miR-155 (as well as decreased levels of miR-141) in breast cancer and of miR-203 in pancreatic cancer correlate with poor survival [121]. Moreover, miRNA can be assessed through relatively noninvasive means from body fluids such as serum, providing an advantage over traditional biopsies [122].

ΔNp63α and ΔNp63α-regulated miRNAs have utility as predictive biomarkers that can help guide personalized treatment plans to aid in the prevention of metastatic cancer. miRNA expression profiles and miRNA biomarkers that correlate with cancerous and normal phenotypes are now routinely identified using Next Generation Sequencing. miRNA that regulate ΔNp63α in EMT may therefore serve as novel therapeutic targets for inhibiting metastasis, either via blockade of metastatic-inducing miRNAs through antagonistic mRNA-mimic complementary base pairing or through exogenous introduction of miRNAs that induce tumor-suppressing effects [123]. Finally, miRNA expression profiles may also have the potential to predict the outcome of a therapeutic regimen [124].

## CONCLUSIONS

There is growing evidence for the involvement of in EMT in the development of metastatic cancers, with implications in breast, lung, prostate, bladder, and gastric cancers. Identifying the mechanisms by which EMT induces a motile and invasive cell phenotype is therefore valuable in combating the various types of malignant carcinoma.

This review presents information from multiple studies detailing the diverse involvement of miRNAs and ΔNp63α in the development of EMT-mediated metastatic cancer. miRNAs have emerged as regulators of key EMT transcripts, whose expression patterns could indicate the genomic balance indicative of EMT. The transcription factor ΔNp63α has been shown to regulate multiple miRNAs in addition to cell signaling pathways, including TGFβ, Wnt, and Notch, involved in EMT, as described throughout this review. Since ΔNp63α expression and activity are altered by these signaling pathways to either promote or antagonize EMT, ΔNp63α-regulated miRNAs will also be altered in a similar manner. Understanding ΔNp63α-regulated miRNA profiles could therefore provide clues as to whether specific cancer types will become migratory and invasive. This highlights the importance of uncovering the regulatory network of miRNAs by ΔNp63α within the context of EMT.

Principally, identification of ΔNp63α-regulated miRNAs as novel biomarkers may provide a powerful tool for the prediction of metastatic potential. Further, Next Generation Sequencing may facilitate generation of a miRNA profile of cells undergoing EMT. With further study, such profiling has the potential to create a more personalized patient prognosis and therapeutic development for treatment of high-risk invasive tumors.

## References

[1] World Health Organization (2015). Cancer.

[2] Lamouille S, Xu J, Derynck R (2014). Molecular mechanisms of epithelial-mesenchymal transition. Nature reviews Molecular cell biology.

[3] Voulgari A, Pintzas A (2009). Epithelial-mesenchymal transition in cancer metastasis: mechanisms, markers and strategies to overcome drug resistance in the clinic. Biochimica et biophysica acta.

[4] Kim HY, Jackson TR, Davidson LA (2016). On the role of mechanics in driving mesenchymal-to-epithelial transitions. Seminars in cell & developmental biology.

[5] Polyak K, Weinberg RA (2009). Transitions between epithelial and mesenchymal states: acquisition of malignant and stem cell traits. Nature reviews Cancer.

[6] Li L, Li W (2015). Epithelial-mesenchymal transition in human cancer: Comprehensive reprogramming of metabolism, epigenetics, and differentiation. Pharmacology & therapeutics.

[7] Tsai JH, Yang J (2013). Epithelial-mesenchymal plasticity in carcinoma metastasis. Genes & development.

[8] Gonzalez DM, Medici D (2014). Signaling mechanisms of the epithelial-mesenchymal transition. Science signaling.

[9] Son H, Moon A (2010). Epithelial-mesenchymal Transition and Cell Invasion. Toxicological research.

[10] Cano A, Perez-Moreno MA, Rodrigo I, Locascio A, Blanco MJ, MG del Barrio, Portillo F, Nieto MA (2000). The transcription factor snail controls epithelial-mesenchymal transitions by repressing E-cadherin expression. Nature cell biology.

[11] Oda H, Tsukita S, Takeichi M (1998). Dynamic behavior of the cadherin-based cell-cell adhesion system during Drosophila gastrulation. Developmental biology.

[12] Casas E, Kim J, Bendesky A, Ohno-Machado L, Wolfe CJ, Yang J (2011). Snail2 is an essential mediator of Twist1-induced epithelial mesenchymal transition and metastasis. Cancer research.

[13] Eger A, Aigner K, Sonderegger S, Dampier B, Oehler S, Schreiber M, Berx G, Cano A, Beug H, Foisner R (2005). DeltaEF1 is a transcriptional repressor of E-cadherin and regulates epithelial plasticity in breast cancer cells. Oncogene.

[14] Miyoshi A, Kitajima Y, Sumi K, Sato K, Hagiwara A, Koga Y, Miyazaki K (2004). Snail and SIP1 increase cancer invasion by upregulating MMP family in hepatocellular carcinoma cells. British journal of cancer.

[15] Yang A, Kaghad M, Wang Y, Gillett E, Fleming MD, Dotsch V, Andrews NC, Caput D, McKeon F (1998). p63, a p53 homolog at 3q27-29, encodes multiple products with transactivating, death-inducing, and dominant-negative activities. Molecular cell.

[16] Vanbokhoven H, Melino G, Candi E, Declercq W (2011). p63, a story of mice and men. The Journal of investigative dermatology.

[17] Serber Z, Lai HC, Yang A, Ou HD, Sigal MS, Kelly AE, Darimont BD, Duijf PH, H Van Bokhoven, McKeon F, Dotsch V (2002). A C-terminal inhibitory domain controls the activity of p63 by an intramolecular mechanism. Molecular and cellular biology.

[18] Yang A, Schweitzer R, Sun D, Kaghad M, Walker N, Bronson RT, Tabin C, Sharpe A, Caput D, Crum C, McKeon F (1999). p63 is essential for regenerative proliferation in limb, craniofacial and epithelial development. Nature.

[19] Mills AA, Zheng B, Wang XJ, Vogel H, Roop DR, Bradley A (1999). p63 is a p53 homologue required for limb and epidermal morphogenesis. Nature.

[20] Carroll DK, Carroll JS, Leong CO, Cheng F, Brown M, Mills AA, Brugge JS, Ellisen LW (2006). p63 regulates an adhesion programme and cell survival in epithelial cells. Nature cell biology.

[21] Olsen JR, Oyan AM, Rostad K, Hellem MR, Liu J, Li L, Micklem DR, Haugen H, Lorens JB, Rotter V, Ke XS, Lin B, Kalland KH (2013). p63 attenuates epithelial to mesenchymal potential in an experimental prostate cell model. PloS one.

[22] Lopardo T, N Lo Iacono, Marinari B, Giustizieri ML, Cyr DG, Merlo G, Crosti F, Costanzo A, Guerrini L (2008). Claudin-1 is a p63 target gene with a crucial role in epithelial development. PloS one.

[23] Higashikawa K, Yoneda S, Tobiume K, Saitoh M, Taki M, Mitani Y, Shigeishi H, Ono S, Kamata N (2009). DeltaNp63alpha-dependent expression of Id-3 distinctively suppresses the invasiveness of human squamous cell carcinoma. International journal of cancer.

[24] Barbieri CE, Tang LJ, Brown KA, Pietenpol JA (2006). Loss of p63 leads to increased cell migration and up-regulation of genes involved in invasion and metastasis. Cancer research.

[25] Higashikawa K, Yoneda S, Tobiume K, Taki M, Shigeishi H, Kamata N (2007). Snail-induced down-regulation of DeltaNp63alpha acquires invasive phenotype of human squamous cell carcinoma. Cancer research.

[26] Alexandrova EM, Petrenko O, Nemajerova A, Romano RA, Sinha S, Moll UM (2013). DeltaNp63 regulates select routes of reprogramming via multiple mechanisms. Cell death and differentiation.

[27] Laurenzana A, Biagioni A, Bianchini F, Peppicelli S, Chilla A, Margheri F, Luciani C, Pimpinelli N, M Del Rosso, Calorini L, Fibbi G (2015). Inhibition of uPAR-TGFbeta crosstalk blocks MSC-dependent EMT in melanoma cells. Journal of molecular medicine.

[28] Rhodes LV, Tate CR, Segar HC, Burks HE, Phamduy TB, Hoang V, Elliott S, Gilliam D, Pounder FN, Anbalagan M, Chrisey DB, Rowan BG, Burow ME, Collins-Burow BM (2014). Suppression of triple-negative breast cancer metastasis by pan-DAC inhibitor panobinostat via inhibition of ZEB family of EMT master regulators. Breast cancer research and treatment.

[29] Yoon NA, Jo HG, Lee UH, Park JH, Yoon JE, Ryu J, Kang SS, Min YJ, Ju SA, Seo EH, Huh IY, Lee BJ, Park JW (2016). Tristetraprolin suppresses the EMT through the down-regulation of Twist1 and Snail1 in cancer cells. Oncotarget.

[30] Zong H, Yin B, Zhou H, Cai D, Ma B, Xiang Y (2014). Inhibition of mTOR pathway attenuates migration and invasion of gallbladder cancer via EMT inhibition. Molecular biology reports.

[31] Bi WR, Yang CQ, Shi Q (2012). Transforming growth factor-beta1 induced epithelial-mesenchymal transition in hepatic fibrosis. Hepato-gastroenterology.

[32] Gaarenstroom T, Hill CS (2014). TGF-beta signaling to chromatin: how Smads regulate transcription during self-renewal and differentiation. Seminars in cell & developmental biology.

[33] O’Connor JW, Gomez EW (2014). Biomechanics of TGFbeta-induced epithelial-mesenchymal transition: implications for fibrosis and cancer. Clinical and translational medicine.

[34] Thuault S, Tan EJ, Peinado H, Cano A, Heldin CH, Moustakas A (2008). HMGA2 and Smads co-regulate SNAIL1 expression during induction of epithelial-to-mesenchymal transition. The Journal of biological chemistry.

[35] Adorno M, Cordenonsi M, Montagner M, Dupont S, Wong C, Hann B, Solari A, Bobisse S, Rondina MB, Guzzardo V, Parenti AR, Rosato A, Bicciato S, Balmain A, Piccolo S (2009). A Mutant-p53/Smad complex opposes p63 to empower TGFbeta-induced metastasis. Cell.

[36] Balboni AL, Hutchinson JA, DeCastro AJ, Cherukuri P, Liby K, Sporn MB, Schwartz GN, Wells WA, Sempere LF, Yu PB, DiRenzo J (2013). DeltaNp63alpha-mediated activation of bone morphogenetic protein signaling governs stem cell activity and plasticity in normal and malignant mammary epithelial cells. Cancer research.

[37] Balboni AL, Cherukuri P, Ung M, DeCastro AJ, Cheng C, DiRenzo J (2015). p53 and DeltaNp63alpha Coregulate the Transcriptional and Cellular Response to TGFbeta and BMP Signals. Molecular cancer research.

[38] Oh JE, Kim RH, Shin KH, Park NH, Kang MK (2011). DeltaNp63alpha protein triggers epithelial-mesenchymal transition and confers stem cell properties in normal human keratinocytes. The Journal of biological chemistry.

[39] Dang TT, Esparza MA, Maine EA, Westcott JM, Pearson GW (2015). DeltaNp63alpha Promotes Breast Cancer Cell Motility through the Selective Activation of Components of the Epithelial-to-Mesenchymal Transition Program. Cancer research.

[40] Dang TT, Westcott JM, Maine EA, Kanchwala M, Xing C, Pearson GW (2016). DeltaNp63alpha induces the expression of FAT2 and Slug to promote tumor invasion. Oncotarget.

[41] Katoh M, Katoh M (2007). WNT signaling pathway and stem cell signaling network. Clinical cancer research.

[42] Patturajan M, Nomoto S, Sommer M, Fomenkov A, Hibi K, Zangen R, Poliak N, Califano J, Trink B, Ratovitski E, Sidransky D (2002). DeltaNp63 induces beta-catenin nuclear accumulation and signaling. Cancer cell.

[43] Drewelus I, Gopfert C, Hippel C, Dickmanns A, Damianitsch K, Pieler T, Dobbelstein M (2010). p63 antagonizes Wnt-induced transcription. Cell cycle.

[44] Lee KB, Ye S, Park MH, Park BH, Lee JS, Kim SM (2014). p63-Mediated activation of the beta-catenin/c-Myc signaling pathway stimulates esophageal squamous carcinoma cell invasion and metastasis. Cancer letters.

[45] Katoh I, Fukunishi N, Fujimuro M, Kasai H, Moriishi K, Hata R, Kurata S (2016). Repression of Wnt/beta-catenin response elements by p63 (TP63). Cell cycle.

[46] Leonard MK, Kommagani R, Payal V, Mayo LD, Shamma HN, Kadakia MP (2011). DeltaNp63alpha regulates keratinocyte proliferation by controlling PTEN expression and localization. Cell death and differentiation.

[47] Shaw M, Cohen P, Alessi DR (1997). Further evidence that the inhibition of glycogen synthase kinase-3beta by IGF-1 is mediated by PDK1/PKB-induced phosphorylation of Ser-9 and not by dephosphorylation of Tyr-216. FEBS letters.

[48] Wang Z, Li Y, Kong D, Sarkar FH (2010). The role of Notch signaling pathway in epithelial-mesenchymal transition (EMT) during development and tumor aggressiveness. Current drug targets.

[49] Capaccione KM, Pine SR (2013). The Notch signaling pathway as a mediator of tumor survival. Carcinogenesis.

[50] Heitzler P (2010). Biodiversity and noncanonical Notch signaling. Current topics in developmental biology.

[51] Espinoza I, Pochampally R, Xing F, Watabe K, Miele L (2013). Notch signaling: targeting cancer stem cells and epithelial-to-mesenchymal transition. OncoTargets and therapy.

[52] Sen T, Chang X, Sidransky D, Chatterjee A (2010). Regulation of DeltaNp63alpha by NFkappaBeta. Cell cycle.

[53] Yugawa T, Narisawa-Saito M, Yoshimatsu Y, Haga K, Ohno S, Egawa N, Fujita M, Kiyono T (2010). DeltaNp63alpha repression of the Notch1 gene supports the proliferative capacity of normal human keratinocytes and cervical cancer cells. Cancer research.

[54] Tadeu AM, Horsley V (2013). Notch signaling represses p63 expression in the developing surface ectoderm. Development.

[55] Sasaki Y, Ishida S, Morimoto I, Yamashita T, Kojima T, Kihara C, Tanaka T, Imai K, Nakamura Y, Tokino T (2002). The p53 family member genes are involved in the Notch signal pathway. The Journal of biological chemistry.

[56] Zaravinos A (2015). The Regulatory Role of MicroRNAs in EMT and Cancer. Journal of oncology.

[57] Ameres SL, Zamore PD (2013). Diversifying microRNA sequence and function. Nature reviews Molecular cell biology.

[58] Lin SL, Miller JD, Ying SY (2006). Intronic microRNA (miRNA). Journal of biomedicine & biotechnology.

[59] Tucci P, Agostini M, Grespi F, Markert EK, Terrinoni A, Vousden KH, Muller PA, Dotsch V, Kehrloesser S, Sayan BS, Giaccone G, Lowe SW, Takahashi N, Vandenabeele P, Knight RA, Levine AJ (2012). Loss of p63 and its microRNA-205 target results in enhanced cell migration and metastasis in prostate cancer. Proceedings of the National Academy of Sciences of the United States of America.

[60] Tran MN, Choi W, Wszolek MF, Navai N, Lee IL, Nitti G, Wen S, Flores ER, Siefker-Radtke A, Czerniak B, Dinney C, Barton M, McConkey DJ (2013). The p63 protein isoform DeltaNp63alpha inhibits epithelial-mesenchymal transition in human bladder cancer cells: role of MIR-205. The Journal of biological chemistry.

[61] Gregory PA, Bert AG, Paterson EL, Barry SC, Tsykin A, Farshid G, Vadas MA, Khew-Goodall Y, Goodall GJ (2008). The miR-200 family and miR-205 regulate epithelial to mesenchymal transition by targeting ZEB1 and SIP1. Nature cell biology.

[62] Louafi F, Martinez-Nunez RT, Sanchez-Elsner T (2010). MicroRNA-155 targets SMAD2 and modulates the response of macrophages to transforming growth factor-{beta}. The Journal of biological chemistry.

[63] Mattiske S, Ho K, Noll JE, Neilsen PM, Callen DF, Suetani RJ (2013). TAp63 regulates oncogenic miR-155 to mediate migration and tumour growth. Oncotarget.

[64] Qi J, Rice SJ, Salzberg AC, Runkle EA, Liao J, Zander DS, Mu D (2012). MiR-365 regulates lung cancer and developmental gene thyroid transcription factor 1. Cell cycle.

[65] Watanabe S, Ueda Y, Akaboshi S, Hino Y, Sekita Y, Nakao M (2009). HMGA2 maintains oncogenic RAS-induced epithelial-mesenchymal transition in human pancreatic cancer cells. The American journal of pathology.

[66] Thuault S, Valcourt U, Petersen M, Manfioletti G, Heldin CH, Moustakas A (2006). Transforming growth factor-beta employs HMGA2 to elicit epithelial-mesenchymal transition. The Journal of cell biology.

[67] Wang FE, Zhang C, Maminishkis A, Dong L, Zhi C, Li R, Zhao J, Majerciak V, Gaur AB, Chen S, Miller SS (2010). MicroRNA-204/211 alters epithelial physiology. FASEB journal.

[68] Liu YN, Yin JJ, Abou-Kheir W, Hynes PG, Casey OM, Fang L, Yi M, Stephens RM, Seng V, Sheppard-Tillman H, Martin P, Kelly K (2013). MiR-1 and miR-200 inhibit EMT via Slug-dependent and tumorigenesis via Slug-independent mechanisms. Oncogene.

[69] Zhang Z, Zhang B, Li W, Fu L, Fu L, Zhu Z, Dong JT (2011). Epigenetic Silencing of miR-203 Upregulates SNAI2 and Contributes to the Invasiveness of Malignant Breast Cancer Cells. Genes Cancer.

[70] Park SM, Gaur AB, Lengyel E, Peter ME (2008). The miR-200 family determines the epithelial phenotype of cancer cells by targeting the E-cadherin repressors ZEB1 and ZEB2. Genes & development.

[71] Cano A, Nieto MA (2008). Non-coding RNAs take centre stage in epithelial-to-mesenchymal transition. Trends in cell biology.

[72] Saydam O, Shen Y, Wurdinger T, Senol O, Boke E, James MF, Tannous BA, Stemmer-Rachamimov AO, Yi M, Stephens RM, Fraefel C, Gusella JF, Krichevsky AM, Breakefield XO (2009). Downregulated microRNA-200a in meningiomas promotes tumor growth by reducing E-cadherin and activating the Wnt/beta-catenin signaling pathway. Molecular and cellular biology.

[73] Bracken CP, Gregory PA, Kolesnikoff N, Bert AG, Wang J, Shannon MF, Goodall GJ (2008). A double-negative feedback loop between ZEB1-SIP1 and the microRNA-200 family regulates epithelial-mesenchymal transition. Cancer research.

[74] Dong P, Karaayvaz M, Jia N, Kaneuchi M, Hamada J, Watari H, Sudo S, Ju J, Sakuragi N (2013). Mutant p53 gain-of-function induces epithelial-mesenchymal transition through modulation of the miR-130b-ZEB1 axis. Oncogene.

[75] Kato M, Zhang J, Wang M, Lanting L, Yuan H, Rossi JJ, Natarajan R (2007). MicroRNA-192 in diabetic kidney glomeruli and its function in TGF-beta-induced collagen expression via inhibition of E-box repressors. Proceedings of the National Academy of Sciences of the United States of America.

[76] Liu X, Wang C, Chen Z, Jin Y, Wang Y, Kolokythas A, Dai Y, Zhou X (2011). MicroRNA-138 suppresses epithelial-mesenchymal transition in squamous cell carcinoma cell lines. The Biochemical journal.

[77] Diaz-Lopez A, Moreno-Bueno G, Cano A (2014). Role of microRNA in epithelial to mesenchymal transition and metastasis and clinical perspectives. Cancer management and research.

[78] Siemens H, Jackstadt R, Hunten S, Kaller M, Menssen A, Gotz U, Hermeking H (2011). miR-34 and SNAIL form a double-negative feedback loop to regulate epithelial-mesenchymal transitions. Cell cycle.

[79] Ru P, Steele R, Newhall P, Phillips NJ, Toth K, Ray RB (2012). miRNA-29b suppresses prostate cancer metastasis by regulating epithelial-mesenchymal transition signaling. Molecular cancer therapeutics.

[80] Zhang J, Zhang H, Liu J, Tu X, Zang Y, Zhu J, Chen J, Dong L, Zhang J (2012). miR-30 inhibits TGF-beta1-induced epithelial-to-mesenchymal transition in hepatocyte by targeting Snail1. Biochemical and biophysical research communications.

[81] Moes M, A Le Bechec, Crespo I, Laurini C, Halavatyi A, Vetter G, A Del Sol, Friederich E (2012). A novel network integrating a miRNA-203/SNAI1 feedback loop which regulates epithelial to mesenchymal transition. PloS one.

[82] Lena AM, Shalom-Feuerstein R, Rivetti di Val Cervo P, Aberdam D, Knight RA, Melino G, Candi E (2008). miR-203 represses ‘stemness’ by repressing DeltaNp63. Cell death and differentiation.

[83] Boominathan L (2010). The guardians of the genome (p53, TA-p73, and TA-p63) are regulators of tumor suppressor miRNAs network. Cancer metastasis reviews.

[84] Xie H, Lee L, Scicluna P, Kavak E, Larsson C, Sandberg R, Lui WO (2015). Novel functions and targets of miR-944 in human cervical cancer cells. International journal of cancer.

[85] Ratovitski EA (2013). Tumor Protein p63/microRNA Network in Epithelial Cancer Cells. Current genomics.

[86] Wu N, Sulpice E, Obeid P, Benzina S, Kermarrec F, Combe S, Gidrol X (2012). The miR-17 family links p63 protein to MAPK signaling to promote the onset of human keratinocyte differentiation. PloS one.

[87] Zhang Z, Hou C, Meng F, Zhao X, Zhang Z, Huang G, Chen W, Fu M, Liao W (2015). MiR-455-3p regulates early chondrogenic differentiation via inhibiting Runx2. FEBS letters.

[88] Niu DF, Kondo T, Nakazawa T, Oishi N, Kawasaki T, Mochizuki K, Yamane T, Katoh R (2012). Transcription factor Runx2 is a regulator of epithelial-mesenchymal transition and invasion in thyroid carcinomas. Laboratory investigation.

[89] Stinson S, Lackner MR, Adai AT, Yu N, Kim HJ, O’Brien C, Spoerke J, Jhunjhunwala S, Boyd Z, Januario T, Newman RJ, Yue P, Bourgon R, Modrusan Z, Stern HM, Warming S (2011). TRPS1 targeting by miR-221/222 promotes the epithelial-to-mesenchymal transition in breast cancer. Science signaling.

[90] Ma L, Young J, Prabhala H, Pan E, Mestdagh P, Muth D, Teruya-Feldstein J, Reinhardt F, Onder TT, Valastyan S, Westermann F, Speleman F, Vandesompele J, Weinberg RA (2010). miR-9, a MYC/MYCN-activated microRNA, regulates E-cadherin and cancer metastasis. Nature cell biology.

[91] Liu S, Kumar SM, Lu H, Liu A, Yang R, Pushparajan A, Guo W, Xu X (2012). MicroRNA-9 up-regulates E-cadherin through inhibition of NF-kappaB1-Snail1 pathway in melanoma. The Journal of pathology.

[92] Ma L, Teruya-Feldstein J, Weinberg RA (2007). Tumour invasion and metastasis initiated by microRNA-10b in breast cancer. Nature.

[93] Bourguignon LY, Wong G, Earle C, Krueger K, Spevak CC (2010). Hyaluronan-CD44 interaction promotes c-Src-mediated twist signaling, microRNA-10b expression, and RhoA/RhoC up-regulation, leading to Rho-kinase-associated cytoskeleton activation and breast tumor cell invasion. The Journal of biological chemistry.

[94] Rivetti di Val Cervo P, Lena AM, Nicoloso M, Rossi S, Mancini M, Zhou H, Saintigny G, Dellambra E, Odorisio T, Mahe C, Calin GA, Candi E, Melino G (2012). p63-microRNA feedback in keratinocyte senescence. Proceedings of the National Academy of Sciences of the United States of America.

[95] L Rodriguez Calleja, Jacques C, Lamoureux F, Baud’huin M, M Tellez Gabriel, Quillard T, Sahay D, Perrot P, Amiaud J, Charrier C, Brion R, Lecanda F, Verrecchia F, Heymann D, Ellisen LW, Ory B (2016). DeltaNp63alpha Silences a miRNA Program to Aberrantly Initiate a Wound-Healing Program That Promotes TGFbeta-Induced Metastasis. Cancer research.

[96] Boominathan L (2010). The tumor suppressors p53, p63, and p73 are regulators of microRNA processing complex. PloS one.

[97] Chakravarti D, Su X, Cho MS, Bui NH, Coarfa C, Venkatanarayan A, Benham AL, RE Flores Gonzalez, Alana J, Xiao W, Leung ML, Vin H, Chan IL, Aquino A, Muller N, Wang H (2014). Induced multipotency in adult keratinocytes through down-regulation of DeltaNp63 or DGCR8. Proceedings of the National Academy of Sciences of the United States of America.

[98] Diao Y, Guo X, Jiang L, Wang G, Zhang C, Wan J, Jin Y, Wu Z (2014). miR-203, a tumor suppressor frequently down-regulated by promoter hypermethylation in rhabdomyosarcoma. The Journal of biological chemistry.

[99] Yi R, Poy MN, Stoffel M, Fuchs E (2008). A skin microRNA promotes differentiation by repressing ‘stemness’. Nature.

[100] Sharifi M, Salehi R, Gheisari Y, Kazemi M (2013). Inhibition of MicroRNA miR-92a Inhibits Cell Proliferation in Human Acute Promyelocytic Leukemia. Turkish journal of haematology.

[101] Fujii T, Shimada K, Tatsumi Y, Hatakeyama K, Obayashi C, Fujimoto K, Konishi N (2015). microRNA-145 promotes differentiation in human urothelial carcinoma through down-regulation of syndecan-1. BMC cancer.

[102] Luo ZL, Luo HJ, Fang C, Cheng L, Huang Z, Dai R, Li K, Tian FZ, Wang T, Tang LJ (2016). Negative correlation of ITCH E3 ubiquitin ligase and miRNA-106b dictates metastatic progression in pancreatic cancer. Oncotarget.

[103] Melino S, Bellomaria A, Nepravishta R, Paci M, Melino G (2014). p63 threonine phosphorylation signals the interaction with the WW domain of the E3 ligase Itch. Cell cycle.

[104] Dong P, Kaneuchi M, Watari H, Sudo S, Sakuragi N (2014). MicroRNA-106b modulates epithelial-mesenchymal transition by targeting TWIST1 in invasive endometrial cancer cell lines. Molecular carcinogenesis.

[105] Galli F, Rossi M, D’Alessandra Y, M De Simone, Lopardo T, Haupt Y, Alsheich-Bartok O, Anzi S, Shaulian E, Calabro V, G La Mantia, Guerrini L (2010). MDM2 and Fbw7 cooperate to induce p63 protein degradation following DNA damage and cell differentiation. Journal of cell science.

[106] Lerner M, Lundgren J, Akhoondi S, Jahn A, Ng HF, F Akbari Moqadam, JA Oude Vrielink, Agami R, ML Den Boer, Grander D, Sangfelt O (2011). MiRNA-27a controls FBW7/hCDC4-dependent cyclin E degradation and cell cycle progression. Cell cycle.

[107] Li J, Wang Y, Song Y, Fu Z, Yu W (2014). miR-27a regulates cisplatin resistance and metastasis by targeting RKIP in human lung adenocarcinoma cells. Molecular cancer.

[108] Ma J, Fang B, Zeng F, Ma C, Pang H, Cheng L, Shi Y, Wang H, Yin B, Xia J, Wang Z (2015). Down-regulation of miR-223 reverses epithelial-mesenchymal transition in gemcitabine-resistant pancreatic cancer cells. Oncotarget.

[109] Xu Y, Sengupta T, Kukreja L, Minella AC (2010). MicroRNA-223 regulates cyclin E activity by modulating expression of F-box and WD-40 domain protein 7. The Journal of biological chemistry.

[110] Zuo K, Li M, Zhang X, Lu C, Wang S, Zhi K, He B (2015). MiR-21 suppresses endothelial progenitor cell proliferation by activating the TGFbeta signaling pathway via downregulation of WWP1. International journal of clinical and experimental pathology.

[111] Odar K, Bostjancic E, Gale N, Glavac D, Zidar N (2012). Differential expression of microRNAs miR-21, miR-31, miR-203, miR-125a-5p and miR-125b and proteins PTEN and p63 in verrucous carcinoma of the head and neck. Histopathology.

[112] Perl AK, Wilgenbus P, Dahl U, Semb H, Christofori G (1998). A causal role for E-cadherin in the transition from adenoma to carcinoma. Nature.

[113] Chen J, Imanaka N, Chen J, Griffin JD (2010). Hypoxia potentiates Notch signaling in breast cancer leading to decreased E-cadherin expression and increased cell migration and invasion. British journal of cancer.

[114] Korsching E, Packeisen J, Liedtke C, Hungermann D, Wulfing P, van Diest PJ, Brandt B, Boecker W, Buerger H (2005). The origin of vimentin expression in invasive breast cancer: epithelial-mesenchymal transition, myoepithelial histogenesis or histogenesis from progenitor cells with bilinear differentiation potential?. The Journal of pathology.

[115] Deyoung MP, Ellisen LW (2007). p63 and p73 in human cancer: defining the network. Oncogene.

[116] C Monroig-Bosque Pdel, Rivera CA, Calin GA (2015). MicroRNAs in cancer therapeutics: “from the bench to the bedside”. Expert opinion on biological therapy.

[117] Steinestel K, Eder S, Schrader AJ, Steinestel J (2014). Clinical significance of epithelial-mesenchymal transition. Clinical and translational medicine.

[118] Jie D, Zhongmin Z, Guoqing L, Sheng L, Yi Z, Jing W, Liang Z (2013). Positive expression of LSD1 and negative expression of E-cadherin correlate with metastasis and poor prognosis of colon cancer. Digestive diseases and sciences.

[119] Whiteland H, Spencer-Harty S, Thomas DH, Davies C, Morgan C, Kynaston H, Bose P, Fenn N, Lewis PD, Bodger O, Jenkins S, Doak SH (2013). Putative prognostic epithelial-to-mesenchymal transition biomarkers for aggressive prostate cancer. Experimental and molecular pathology.

[120] Micalizzi DS, Farabaugh SM, Ford HL (2010). Epithelial-mesenchymal transition in cancer: parallels between normal development and tumor progression. Journal of mammary gland biology and neoplasia.

[121] Greither T, Grochola LF, Udelnow A, Lautenschlager C, Wurl P, Taubert H (2010). Elevated expression of microRNAs 155, 203, 210 and 222 in pancreatic tumors is associated with poorer survival. International journal of cancer.

[122] Xu L, Qi X, Duan S, Xie Y, Ren X, Chen G, Yang X, Han L, Dong Q (2014). MicroRNAs: potential biomarkers for disease diagnosis. Bio-medical materials and engineering.

[123] Kothari AN, Mi Z, Zapf M, Kuo PC (2014). Novel clinical therapeutics targeting the epithelial to mesenchymal transition. Clinical and translational medicine.

[124] Hayes J, Peruzzi PP, Lawler S (2014). MicroRNAs in cancer: biomarkers, functions and therapy. Trends in molecular medicine.

[125] Kim T, Veronese A, Pichiorri F, Lee TJ, Jeon YJ, Volinia S, Pineau P, Marchio A, Palatini J, Suh SS, Alder H, Liu CG, Dejean A, Croce CM (2011). p53 regulates epithelial-mesenchymal transition through microRNAs targeting ZEB1 and ZEB2. The Journal of experimental medicine.

[126] Han F, Wu Y, Jiang W (2015). MicroRNA-18a Decreases Choroidal Endothelial Cell Proliferation and Migration by Inhibiting HIF1A Expression. Medical science monitor.

[127] Boufraqech M, Nilubol N, Zhang L, Gara SK, Sadowski SM, Mehta A, He M, Davis S, Dreiling J, Copland JA, Smallridge RC, Quezado MM, Kebebew E (2015). miR30a inhibits LOX expression and anaplastic thyroid cancer progression. Cancer research.

[128] Peng R, Zhou L, Zhou Y, Zhao Y, Li Q, Ni D, Hu Y, Long Y, Liu J, Lyu Z, Mao Z, Yuan Y, Huang L, Zhao H, Li G, Zhou Q (2015). MiR-30a Inhibits the Epithelial—Mesenchymal Transition of Podocytes through Downregulation of NFATc3. International journal of molecular sciences.

[129] Ory B, Ramsey MR, Wilson C, Vadysirisack DD, Forster N, Rocco JW, Rothenberg SM, Ellisen LW (2011). A microRNA-dependent program controls p53-independent survival and chemosensitivity in human and murine squamous cell carcinoma. The Journal of clinical investigation.

[130] Fang W, Guo J, Cao Y, Wang S, Pang C, Li M, Dou L, Man Y, Huang X, Shen T, Li J (2016). MicroRNA-20a-5p contributes to hepatic glycogen synthesis through targeting p63 to regulate p53 and PTEN expression. Journal of cellular and molecular medicine.

[131] Kim K, Madak-Erdogan Z, Ventrella R, Katzenellenbogen BS (2013). A MicroRNA196a2* and TP63 circuit regulated by estrogen receptor-alpha and ERK2 that controls breast cancer proliferation and invasiveness properties. Hormones & cancer.

[132] B de Melo Maia, Rodrigues IS, Akagi EM, Soares do Amaral N, Ling H, Monroig P, Soares FA, Calin GA, Rocha RM (2016). MiR-223-5p works as an oncomiR in vulvar carcinoma by TP63 suppression. Oncotarget.

[133] Manni I, Artuso S, Careccia S, Rizzo MG, Baserga R, Piaggio G, Sacchi A (2009). The microRNA miR-92 increases proliferation of myeloid cells and by targeting p63 modulates the abundance of its isoforms. FASEB journal.

[134] Sharifi M, Salehi R, Gheisari Y, Kazemi M (2014). Inhibition of microRNA miR-92a induces apoptosis and inhibits cell proliferation in human acute promyelocytic leukemia through modulation of p63 expression. Molecular biology reports.

[135] Nam RK, Benatar T, Wallis CJ, Amemiya Y, Yang W, Garbens A, Naeim M, Sherman C, Sugar L, Seth A (2016). MiR-301a regulates E-cadherin expression and is predictive of prostate cancer recurrence. The Prostate.

